# DEPDC1 and KIF4A synergistically inhibit the malignant biological behavior of osteosarcoma cells through Hippo signaling pathway

**DOI:** 10.1186/s13018-023-03572-4

**Published:** 2023-02-27

**Authors:** Mingming Yang, Hang Zhang, Shichang Gao, Wei Huang

**Affiliations:** Department of Orthopedics, The First Affiliated Hospital of Chongqing Medical University, No. 1 YouYi Road, Yuan Jia Gang, Yu Zhong District, Chongqing, 400016 People’s Republic of China

**Keywords:** DEPDC1, KIF4A, Osteosarcoma cells, Hippo signaling pathway

## Abstract

The treatment of osteosarcoma (OS) is still mainly surgery combined with systematic chemotherapy, and gene therapy is expected to improve the survival rate of patients. This study aimed to explore the effect of DEP domain 1 protein (DEPDC1) and kinesin super-family protein 4A (KIF4A) in OS and understand its mechanism. Th expression of DEPDC1 and KIF4A in OS cells was detected by RT-PCR and western blot. The viability, proliferation, invasion and migration of OS cells and tube formation of human umbilical vein endothelial cells (HUVECs) after indicated treatment were in turn detected by CCK-8 assay, EdU staining, wound healing assay, transwell assay and tube formation assay. The interaction between DEPDC1 and KIF4A was predicted by STRING and confirmed by co-immunoprecipitation. The expression of epithelial-mesenchymal transition (EMT)-related proteins, tube formation-related proteins and Hippo signaling pathway proteins was detected by western blot. As a result, the expression of DEPDC1 and KIF4A was all increased in U2OS cells. Down-regulation of DEPDC1 suppressed the viability, proliferation, invasion and migration of U2OS cells and tube formation of HUVECs, accompanied by the increased expression of E-cadherin and decreased expression of N-cadherin, Vimentin and VEGF. DEPDC1 was confirmed to be interacted with KIF4A. Upregulation of KIF4A partially reversed the effect of DEPDC1 interference on the above biological behaviors of U2OS cells. Down-regulation of DEPDC1 promoted the expression of p-LATS1 and p-YAP in Hippo signaling pathway, which was reversed by upregulation of KIF4A. In conclusion, down-regulation of DEPDC1 inhibited the malignant biological behavior of OS cells through the activation of Hippo signaling pathway, which could be reversed by upregulation of KIF4A.

## Introduction

Osteosarcoma (OS) is a kind of malignant tumor derived from mesenchymal stem cells and most common in children and adolescents [1]. About 80% of osteosarcomas occur in the long bones of the extremities, most often in the long metaphyseal region around the knee joint [2]. The annual incidence of osteosarcoma is 1–4 per million [3, 4]. With the progress of medical treatment, neoadjuvant chemotherapy can effectively improve the 5-year survival rate of patients, but there are still 30–40% of patients with tumor recurrence and metastasis, especially those with lung metastasis, which often lead to respiratory failure and poor prognosis [5, 6]. Therefore, further research on the pathogenesis of OS will help identify new diagnostic and therapeutic targets, improve the prognosis and improve the survival rate of patients.

The DEP domain 1 protein (DEPDC1) is an oncoprotein containing a DEP domain, which has not been detected in 24 normal human tissues, including normal lung tissue, except testicular surface [7–9] and was first reported in bladder cancer [10]. Studies have shown that DEPDC1 protein is involved in a variety of cell functions, such as promoting cell proliferation and inhibiting apoptosis [11–13]. The expression of DEPDC1 is obviously upregulated in some cancers, and the high expression level of DEPDC1 is closely related to the progression of cancer, including hepatocellular carcinoma [14], bladder cancer [15], lung adenocarcinoma [16] and gastric cancer [17]. A current study has indicated that DEPDC1, one of the hub genes, is highly expressed in osteosarcoma, and its high expression is associated with poor prognosis [18]. At present, its specific role and mechanism in OS have not been studied.

Dysregulation of kinesin super-family protein 4A (KIF4A) expression can induce mitosis and aneuploid cell formation [19]. The high expression of KIF4A could be used as a diagnostic and prognostic marker of OS, and the silencing of KIF4A could inhibit the invasion and migration of OS cells, and induce their apoptosis and cell cycle arrest [20]. High expression of KIF4A in OS predicted poor prognosis and promoted tumor growth by activating the MAPK pathway [21]. KIF4A promoted cell proliferation and migration of esophageal squamous cell carcinoma through Hippo signaling pathway [22]. Hippo signaling pathway was also affected by the changes in gene expression and DNA methylation in cholangiocarcinoma [23]. Hippo signaling pathway was involved in the promotion effect on cell proliferation and invasion of OS [24].

Therefore, this study aimed to explore the effect of DEPDC1 and KIF4A in OS and understand its mechanism.

## Materials and methods

### Cell culture and transfection

hFOB1.19, U2OS, SaOS-2 and MG-63 cells were obtained from Procell (Wuhan, China). Human umbilical vein endothelial cells (HUVECs) were provided from American Type Culture Collection (ATCC). All cells were cultivated within the Dulbecco’s Modified Eagle Medium (DMEM) containing 10% fetal bovine serum (FBS) at 37 °C in an incubator under 5% CO_2_.

To knock down DEPDC1 expression and overexpress KIF4A expression in osteosarcoma cells, short hairpin (sh)RNA targeting ANGPT2 (sh-DEPDC1#1 and sh-DEPDC1#2), pcDNA3.1-KIF4A as well as corresponding negative control (sh-NC) and pcDNA3.1-NC were obtained from RIBIO (Guangzhou, China). When OS cells were reached to about 80% confluence, OS cells were transfected with different vectors using Lipofectamine 3000 according to the manufacturer’s protocol. The transfection efficiency was confirmed by RT-qPCR and western blot 48 h later.

### RT-qPCR

After indicated treatment, total RNA from cells was extracted using Trizol reagent (Invitrogen), and the PrimeScript® RT reagent Kit (Takara) was used to make RNA transcribed into cDNA according to the manufacturer’s instructions. Quantitative real-time PCR (RT-qPCR) was performed with SYBR Green qPCR Master Mix using a 7500 Thermocycler (Applied Biosystems). The relative expression levels of DEPDC1 and KIF4A normalized to GAPDH were calculated by the 2^−ΔΔCt^ method [25].

### Western blot

The treated cells were lysed with ice-cold RIPA buffer for 30 min (Beyotime) to obtain the proteins, concentration of which was determined by a bicinchoninic acid protein assay kit (Beyotime). Proteins (20 μg) were isolated with sodium dodecyl sulfate–polyacrylamide gels electrophoresis (SDS-PAGE) and then transferred to a polyvinylidene fluoride (PVDF) membrane (Millipore, Beford, MA, USA). After blocked with 5% nonfat milk at room temperature, the membrane was incubated with the primary antibodies including DEPDC1, PCNA, E-cadherin, N-cadherin, Vimentin, VEGF, KIF4A, p-LATS1, LATS1, p-YAP, YAP, p-MST1, MST1 and GAPDH overnight at 4 °C and then incubated with specific horseradish peroxidase (HRP)-conjugated secondary antibody at room temperature for 1 h. Finally, the proteins were detected using enhanced chemiluminescence reagents and bands density was analyzed by Image J 1.8.0 software.

### CCK-8 assay

Cells (1 × 10^5^ cells/well) were seeded into the 96-well plates and cultured for 24 h. After respective treatment for 24, 48 and 72 h, cells were treated with CCK8 solution (10 μL) and incubated at room temperature for another 4 h. Finally, the absorbance was determined at 450 nm using a microplate reader.

### EdU staining

Cells received respective treatment and seeded into the 96-well plate (1 × 10^5^ cells/well) for incubation about 24 h. Then, cells were incubated with EdU labeling agent for another 4 h. Next, cells were fixed with 4% paraformaldehyde for 15 min at room temperature and then incubated with 0.5% Triton X-100 in PBS for 15 min at room temperature. After PBS washing, cells were stained with anti-EdU working solution at room temperature for 30 min. Finally, cells were incubated with DAPI at room temperature for 30 min, which were then observed by a fluorescence microscope.

### Wound healing assay

After indicated treatment, cells were seeded into the 6‐well plates (5 × 10^4^ cells) and cultured nearly 100% confluence. A sterile plastic micropipette tip was used to scratch a wound along the center line of the well, followed by the incubation for 24 h. The images of the cells were photographed at 0 and 24 h using an optical microscope (Olympus Corporation).

### Transwell assay

After respective treatment, cells were digested by pancreatin and suspended within the serum-free medium. The 200 μL of the cell suspension (5 × 10^3^ cells) was added to the upper chamber coated with Matrigel and the lower chamber was added with 600-μL DMEM containing 10% FBS. After incubation for 24 h at 37 °C, the invaded cells were fixed with 4% formaldehyde fixation and stained with 0.1% crystal violet for 30 min (Beyotime). Subsequently, the invading cells were observed by an optical microscope (Olympus Corporation).

### Tube formation assay

After respective treatment, the supernatant of cells was obtained and used for the incubation of HUVECs in 96-well plates coated with Matrigel for 48 h. The tubes formed by the HUVEC cells were observed by an optical microscope (Olympus Corporation).

### Co-immunoprecipitation

After respective treatment, total proteins in cells were extracted through immunoprecipitation buffer for 30 min on ice. Then, proteins were incubated with anti-DEPDC1 or anti-KIF4A overnight at 4 °C, followed by incubation with protein A/G magnetic beads (Thermo Scientific) at 4 °C for another 3 h. Subsequently, bead-antibody complexes were washed by immunoprecipitation buffer. The above mixture was then centrifuged at 3000*g* to collect the immunoprecipitates, which was analyzed by western blot.

### Statistical analysis

All data were presented as mean ± SD and GraphPad Prism 8.0.1 software was used to analyze the experimental data. A student’s t test was performed for comparisons between two groups and ANOVA followed by Tukey's post hoc test was carried out for comparisons among multiple groups. *P* < 0.05 was considered as statistical significance.

## Results

### DEPDC1 was highly expressed in OS cells, and down-regulation of DEPDC1 inhibited cell proliferation.

**Fig. 1:**
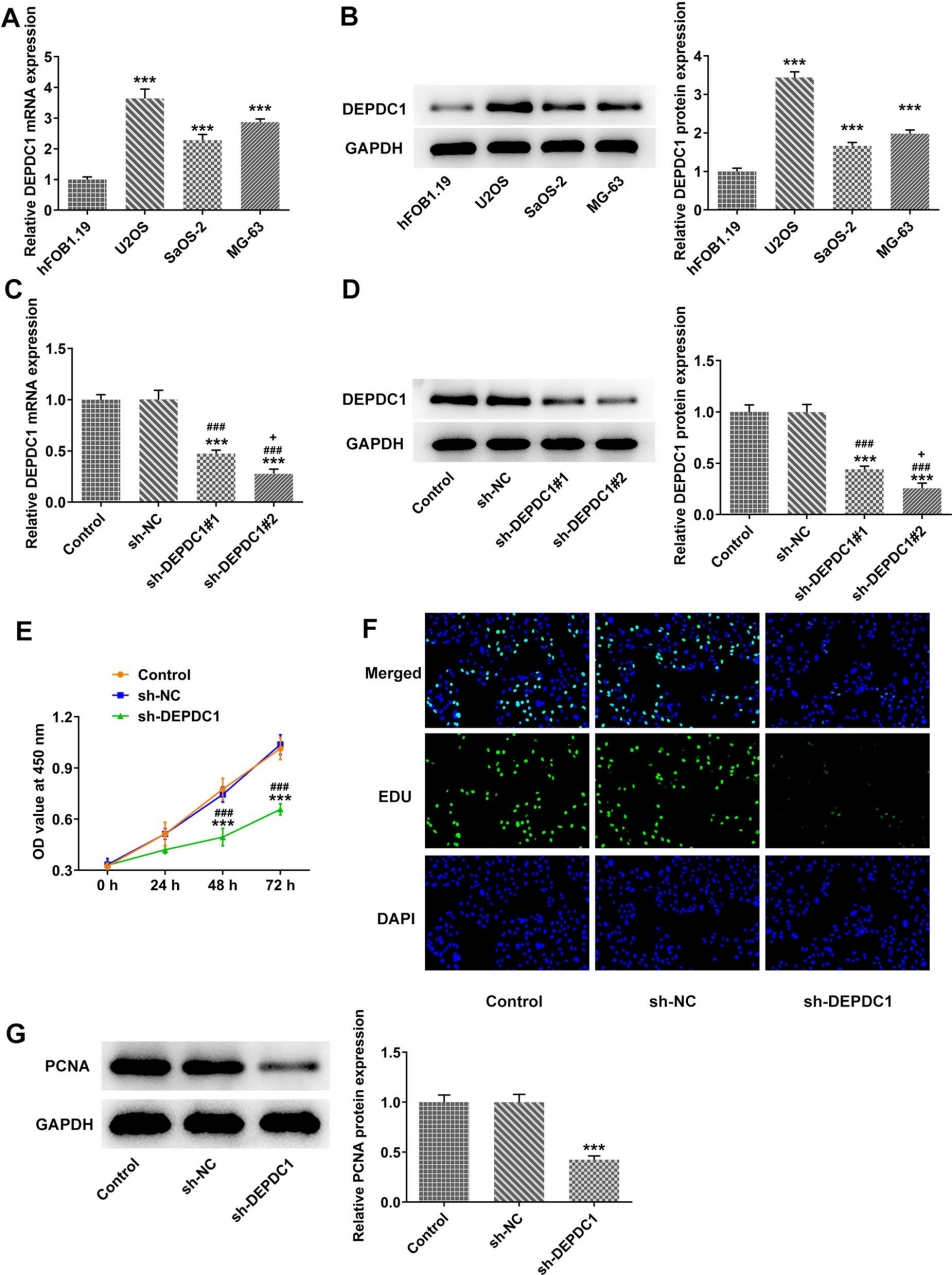
DEPDC1 was highly expressed in OS cells, and down-regulation of DEPDC1 inhibited cell proliferation. **A**/**B** The expression of DEPDC1 in OS cells was detected by RT-qPCR and western blot. ***P < 0.001 versus hFOB1.19 group. **C**/**D** The transfection effects of sh-DEPDC1#1 and sh-DEPDC1#2 in U2OS cells was confirmed by RT-qPCR and western blot. **E** The viability of U2OS cells transfected with si-DEPDC1 was determined by CCK-8 assay. **F** The proliferation of U2OS cells transfected with sh-DEPDC1 was observed by EdU staining. **G** The expression of PCNA in U2OS cells transfected with sh-DEPDC1 was detected by western blot. ***P < 0.001 versus Control group. ^###^*P* < 0.001 versus sh-NC group. ^+^*P* < 0.05 versus sh-DEPDC1#1

The expression of DEPDC1 was upregulated in osteosarcoma cells (U2OS, SaOS-2 and MG-63) compared with that in hFOB1.19 group, and the highest DEPDC1 expression was observed in U2OS cells (Fig. 1A and B). After U2OS cells were transfected with sh-DEPDC1#1 and sh-DEPDC1#2, the expression of DEPDC1 was down-regulated, and the lower expression of DEPDC1 was observed in sh-DEPDC1#2 group (Fig. 1C and D). Therefore, sh-DEPDC1#2 was selected for the subsequent experiment. Down-regulation of DEPDC1 suppressed the viability and proliferation of U2OS cells (Fig. 1E and F). The expression of PCNA was also inhibited by down-regulation of DEPDC1 (Fig. 1G).

### Down-regulation of DEPDC1 inhibited metastasis of OS cells.

**Fig. 2:**
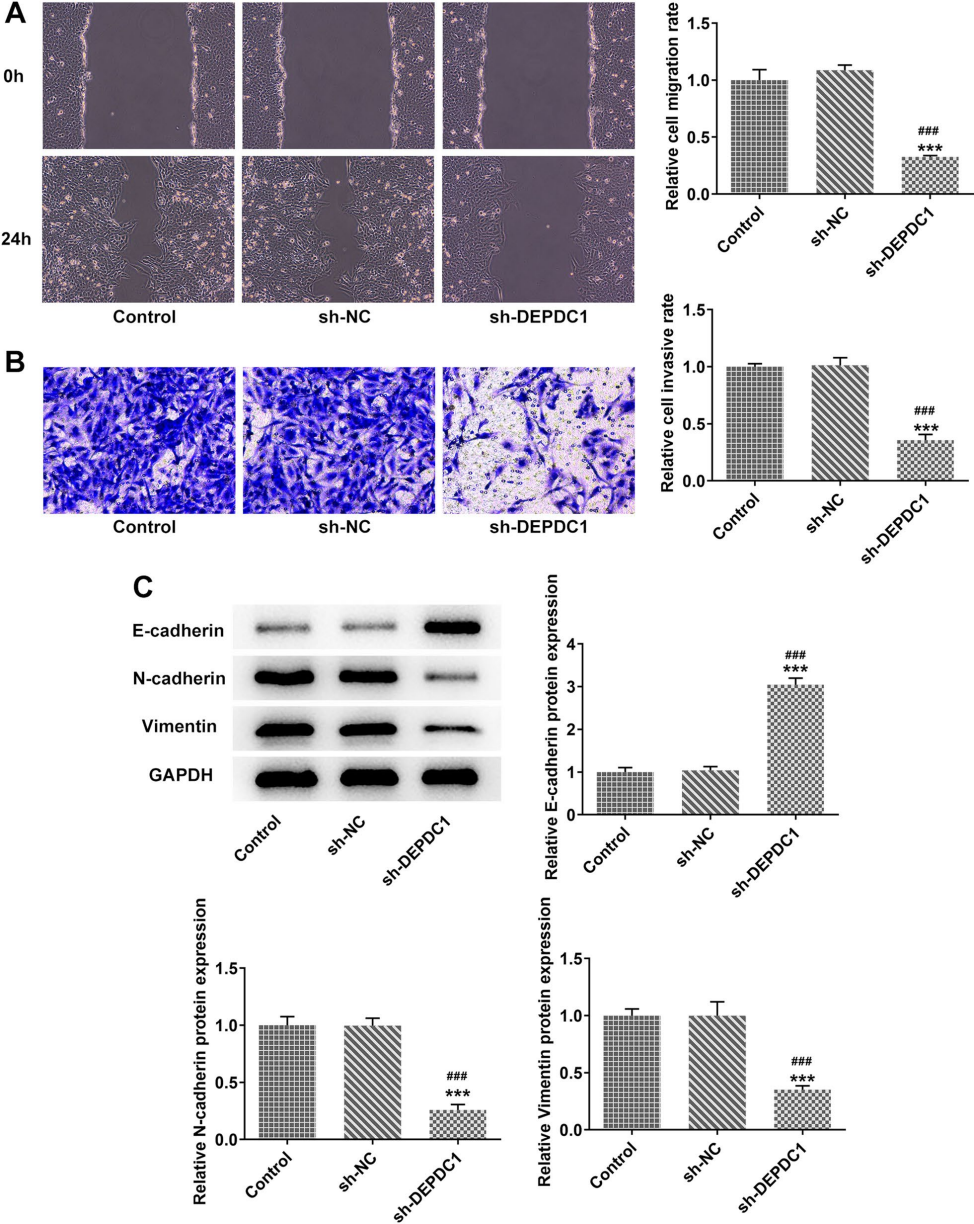
Down-regulation of DEPDC1 inhibited metastasis of OS cells. **A** The migration of U2OS cells transfected with sh-DEPDC1 was detected by wound healing assay. **B** The invasion of U2OS cells transfected with sh-DEPDC1 was detected by transwell assay. **C** Th expression of EMT-related proteins in U2OS cells transfected with sh-DEPDC1 was detected by western blot. ***P < 0.001 versus Control group. ^###^*P* < 0.001 versus sh-NC group

When U2OS cells were transfected with sh-DEPDC1, down-regulation of DEPDC1 inhibited the migration and invasion of U2OS cells (Fig. 2A and B). The expression of E-cadherin was upregulated while the expression of N-cadherin and Vimentin was down-regulated by the down-regulation of DEPDC1 in U2OS cells (Fig. 2C).

### Down-regulation of DEPDC1 inhibited angiogenesis in OS cells.

**Fig. 3:**
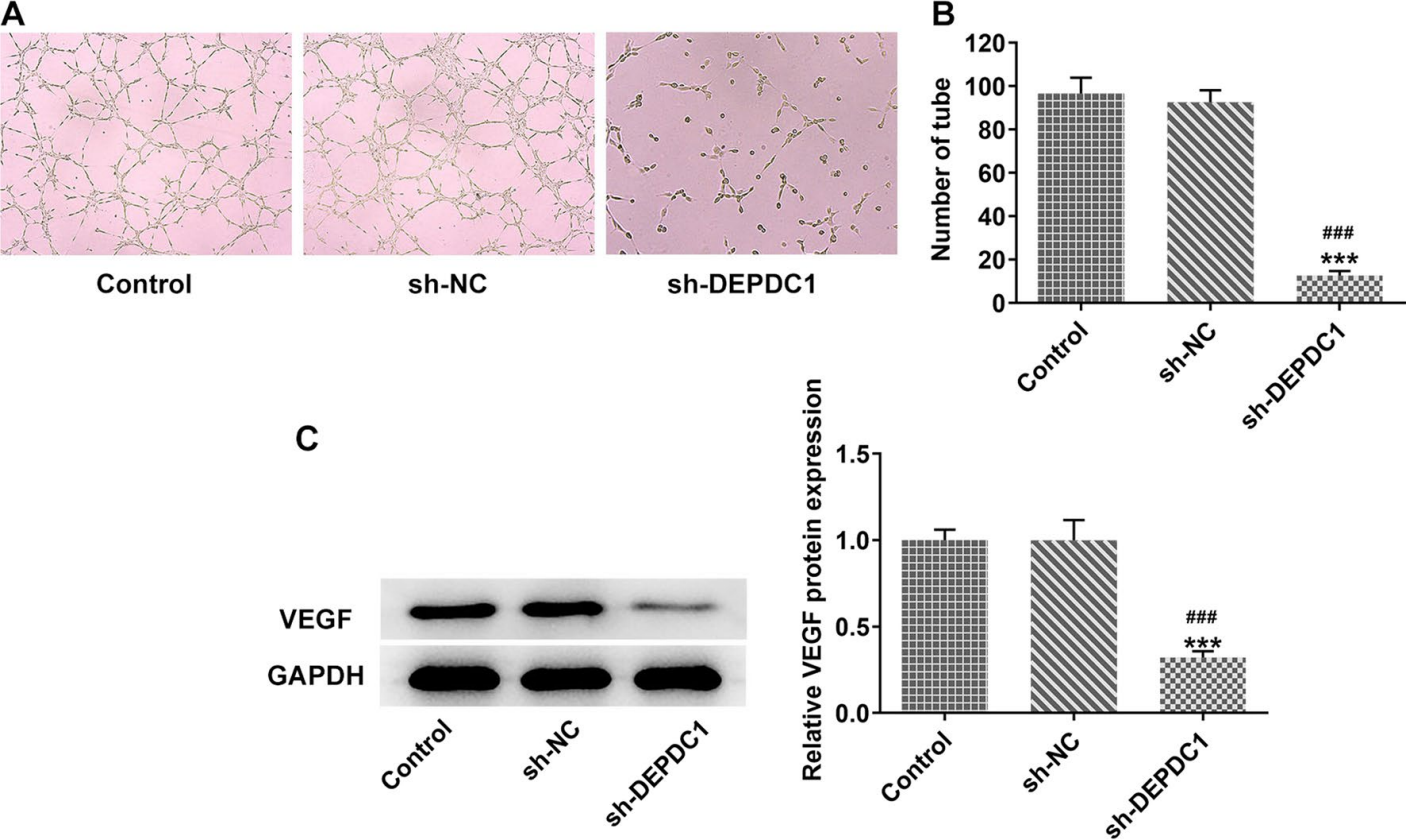
Down-regulation of DEPDC1 inhibited angiogenesis in OS cells. **A**/**B** The number of HUVECs tube formation in the supernatant of U2OS cells transfected with sh-DEPDC1 was analyzed by tube formation assay. **C** The expression of angiogenesis-related protein in U2OS cells transfected with sh-DEPDC1 was detected by western blot. ***P < 0.001 versus Control group. ^###^*P* < 0.001 versus sh-NC group

The images of tube formation of HUVEC are presented in Fig. 3A. The number of tubes was reduced by the down-regulation of DEPDC1 (Fig. 3B). The expression of VEGF was also reduced in U2OS cells transfected with sh-DEPDC1 (Fig. 3C).

### KIF4A was highly expressed in OS cells and interacted with DEPDC1.

**Fig. 4:**
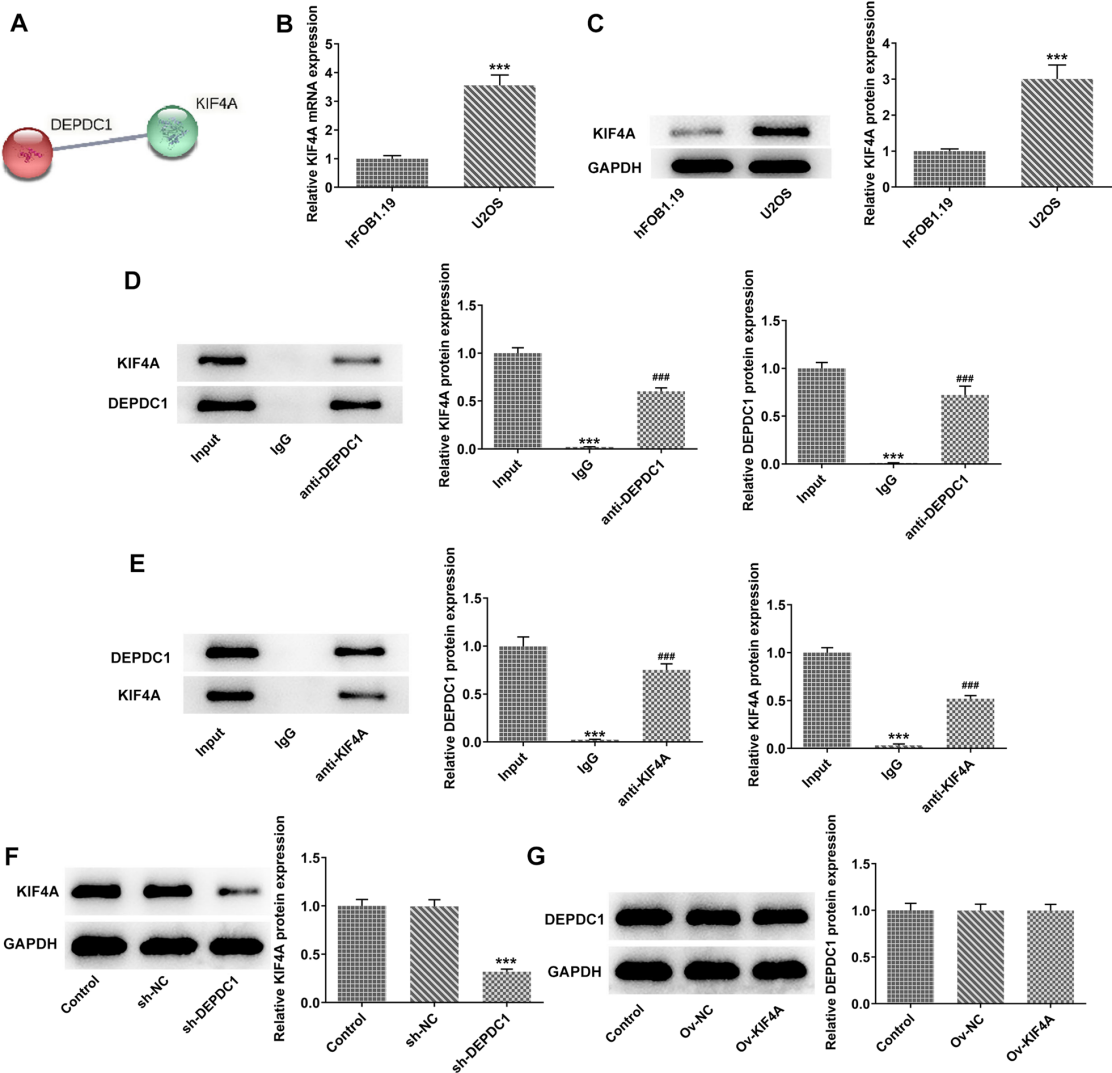
KIF4A was highly expressed in OS cells and interacted with DEPDC1. **A** The interaction between DEPDC1 and KIF4A was confirmed by STRING. **B**/**C** The expression of KIF4A in U2OS cells was detected by RT-qPCR and western blot. ***P < 0.001 versus hFOB1.19 group. **D** The expression of KIF4A and DEPDC1 in proteins incubating with anti-DEPDC1 was detected by western blot. **E** The expression of KIF4A and DEPDC1 in proteins incubating with anti-KIF4A was detected by western blot. ***P < 0.001 versus Input group. ^###^*P* < 0.001 versus Input group. **F** The expression of KIF4A in U2OS cells transfected with sh-DEPDC1 was detected by western blot. ***P < 0.001 versus Control group. **G** The expression of DEPDC1 in U2OS cells transfected with Ov-KIF4A was detected by western blot

STRING found a potential interaction between DEPDC1 and KIF4A (Fig. 4A). Th expression of KIF4A in U2OS cells was higher than that in hFOB1.19 group (Fig. 4B and C). The expression of KIF4A or DEPDC1 in U2OS cells was observed when the protein samples were incubated with anti-DEPDC1 or anti-KIF4A, which indicating that DEPDC1 could interact with KIF4A (Fig. 4D and E). Down-regulation of DEPDC1 suppressed the KIF4A expression, while KIF4A overexpression had no obvious effect on DEPDC1 expression (Fig. 4F and G).

### Upregulation of KIF4A partially reversed the effect of DEPDC1 interference on proliferation of OS cells

**Fig. 5:**
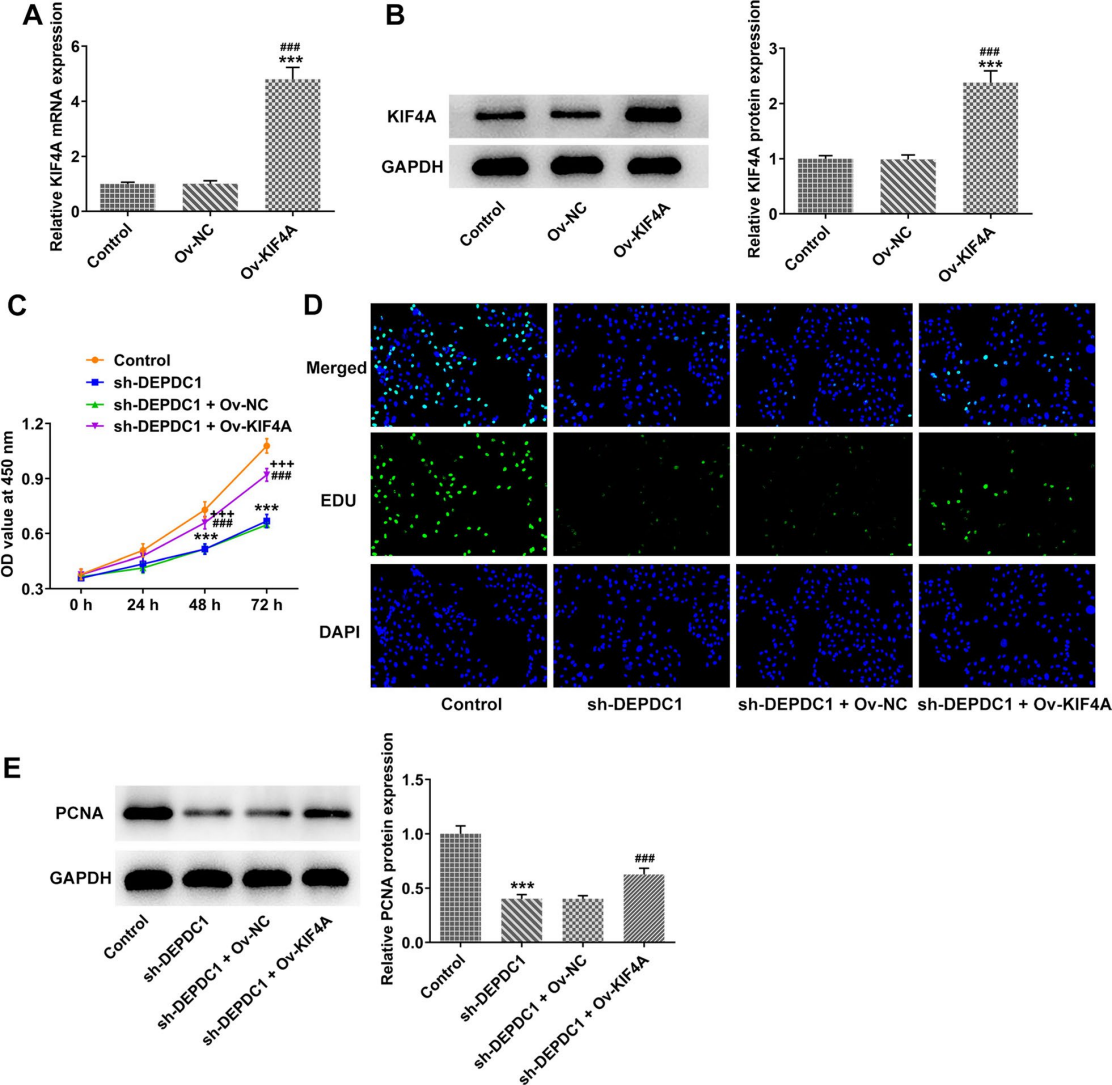
Upregulation of KIF4A partially reversed the effect of DEPDC1 interference on proliferation of OS cells. **A**/**B** The expression of KIF4A in U2OS cells transfected with Ov-KIF4A was detected by RT-qPCR and western blot. ***P < 0.001 versus Control group. ^###^*P* < 0.001 versus Ov-NC group. **C** The viability of U2OS cells transfected with sh-DEPDC1 and Ov-KIF4A was determined by CCK-8 assay. **D** The proliferation of U2OS cells transfected with si-DEPDC1 and Ov-KIF4A was observed by EdU staining. **E** The expression of PCNA in U2OS cells transfected with sh-DEPDC1 and Ov-KIF4A was detected by western blot. ***P < 0.001 versus Control group. ^###^*P* < 0.001 versus sh-NC group. ^+++^*P* < 0.001 versus si-DEPDC1 + Ov-NC group

The expression of KIF4A was upregulated in U2OS cells transfected with Ov-KIF4A (Fig. 5A and B). Upregulation of KIF4A improved the decreased viability and proliferation of U2OS cells induced by the down-regulation of DEPDC1 (Fig. 5C and D). The expression of PCNA was upregulated in U2OS cells co-transfected with sh-DEPDC1 and Ov-KIF4A (Fig. 5E).

### Upregulation of KIF4A partially reversed the effect of DEPDC1 interference on metastasis and angiogenesis of OS cells

**Fig. 6:**
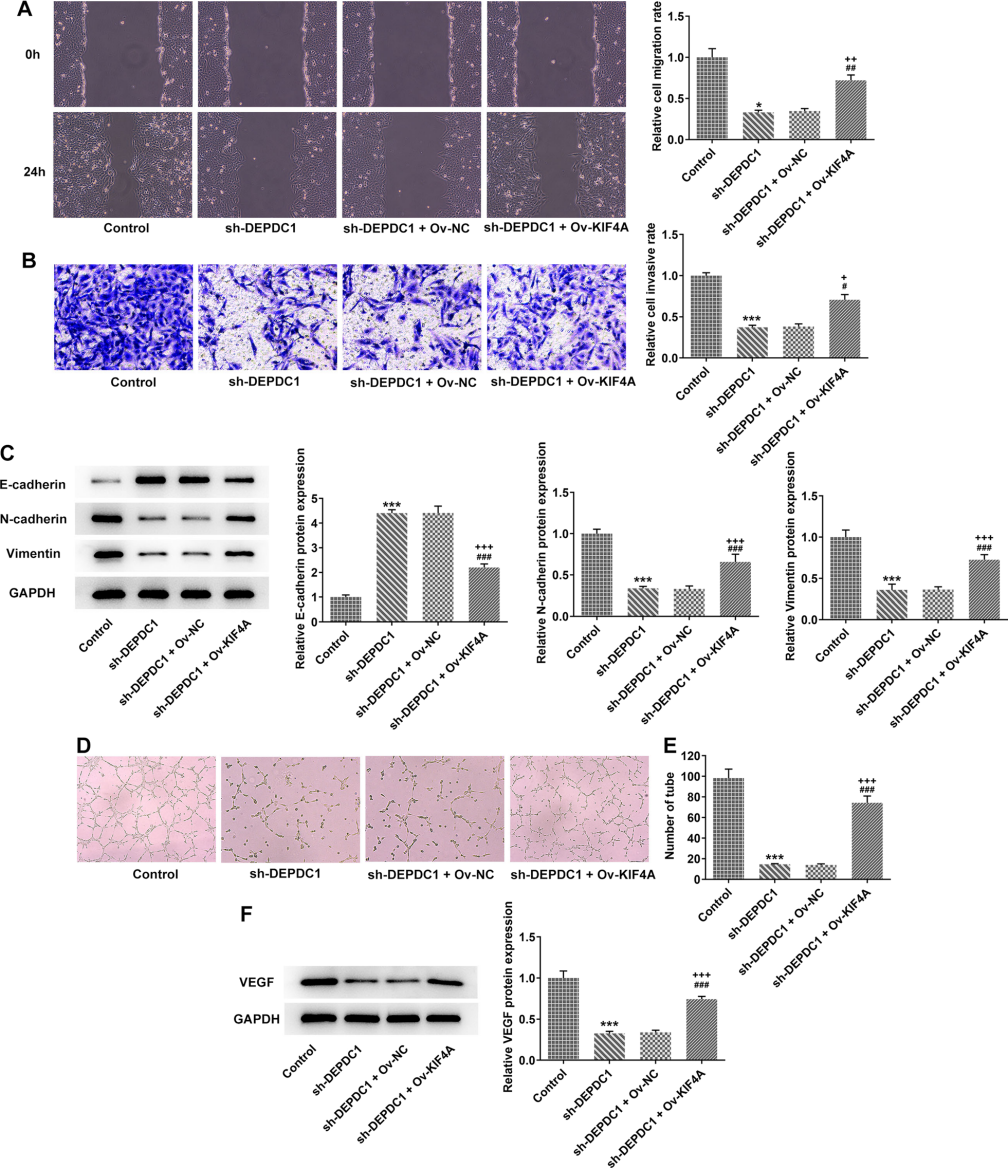
Upregulation of KIF4A partially reversed the effect of DEPDC1 interference on metastasis and angiogenesis of OS cells. **A** The migration of U2OS cells transfected with sh-DEPDC1 and Ov-KIF4A was detected by wound healing assay. **B** The invasion of U2OS cells transfected with si-DEPDC1 and Ov-KIF4A was detected by transwell assay. **C** Th expression of EMT-related proteins in U2OS cells transfected with sh-DEPDC1 and Ov-KIF4A was detected by western blot. **D**/**E** The number of HUVECs tube formation in the supernatant of U2OS cells transfected with sh-DEPDC1 and Ov-KIF4A was analyzed by tube formation assay. **F** The expression of angiogenesis-related protein in U2OS cells transfected with si-DEPDC1 and Ov-KIF4A was detected by western blot. *P < 0.05 and ***P < 0.001 versus Control group. ^##^*P* < 0.01 and ^###^*P* < 0.001 versus sh-NC group. ^++^*P* < 0.01 and ^+++^*P* < 0.001 versus sh-DEPDC1 + Ov-NC group

Upregulation of KIF4A promoted the migration and invasion of U2OS cells transfected with sh-DEPDC1 (Fig. 6A and B) by decreasing the expression of E-cadherin and increasing the expression of N-cadherin and Vimentin (Fig. 6C). Upregulation of KIF4A also increased the number of tube and promoted the expression of VEGF in U2OS cells transfected with sh-DEPDC1 (Fig. 6D–F).

### DEPDC1 and KIF4A synergistically regulated Hippo signaling pathway

**Fig. 7:**
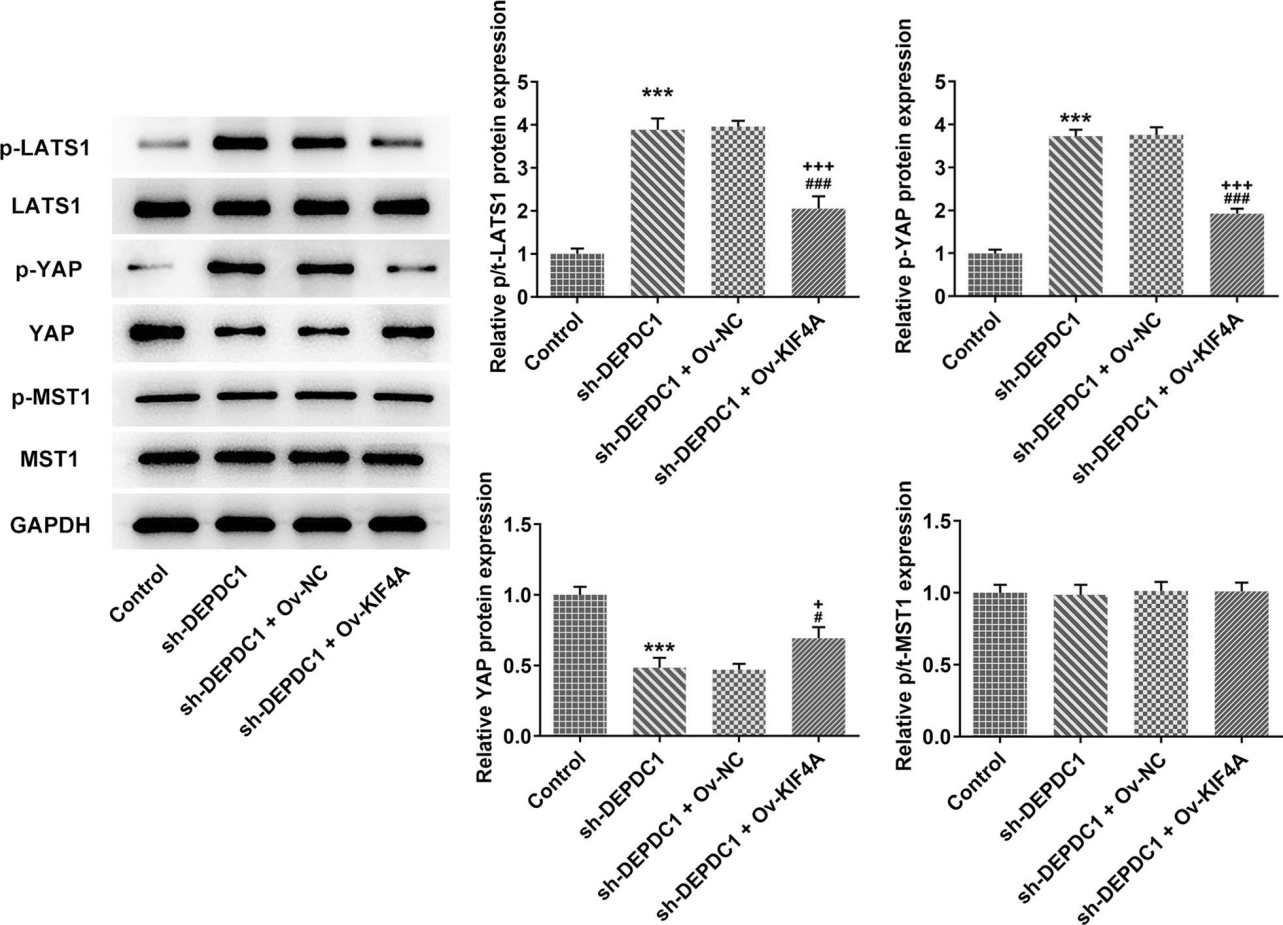
DEPDC1 and KIF4A synergistically regulated Hippo signaling pathway. Th expression of Hippo signaling pathway-related proteins in in U2OS cells transfected with sh-DEPDC1 and Ov-KIF4A was detected by western blot. ***P < 0.001 versus Control group. ^###^*P* < 0.001 versus sh-NC group. ^+++^*P* < 0.001 versus sh-DEPDC1 + Ov-NC group

Down-regulation of DEPDC1 increased the expression of p-LATS1 and p-YAP, while decreased the YAP expression in U2OS cells, which was reversed by upregulation of KIF4A. The expression of LATS1, p-MST1 and MST1 was not obviously changed in U2OS cells whether or not receiving transfection (Fig. 7).

## Discussion

In the current study, we found that DEPDC1 was upregulated in OS cell lines. The down-regulation of DEPDC1 inhibited the viability, migration, invasion, and proliferation of OS cells and tube formation of HUVECs. Our further study clarified that DEPDC1 was interacted with KIF4A. Upregulation of KIF4A could weaken the effect of DEPDC1 interference to improve the viability, migration, invasion, and proliferation of OS cells and tube formation of HUVECs.

DEPDC1 has been shown to be highly expressed in most tumors. Amisaki et al. found that DEPDC1 expression was upregulated in hepatocellular carcinoma tissues compared with normal livers, and the high expression of DEPDC1 in tumor tissues was associated with tumor progression and poor prognosis [26]. DEPDC1 was reported to be overexpressed at both mRNA and protein levels in nasopharyngeal carcinoma tissues compared with normal or non-tumor tissues, and the siRNA-mediated deletion of DEPDC1 significantly inhibited the proliferation of nasopharyngeal carcinoma cell lines CNE-1 and HNE-1 [27]. DEPDC1 interference suppressed hepatocellular carcinoma cell proliferation, colony formation and invasion in vitro and HUVEC angiogenesis [28]. Overexpression of DEPDC1-induced EMT of HepG2 cells with the upregulated expression of N-cadherin and Vimentin, and down-regulated expression of E-cadherin and Slug and promoted the capillary tubule formation [14]. In this study, we found that DEPDC1 expression was increased in OS cell lines. After knockdown of DEPDC1, the proliferation, invasion and migration of U2OS cells and HUVEC tube formation were all inhibited obviously. Previous studies indicated that enhanced KIF4A expression predicted poor prognosis and promoted tumor growth in OS and down-regulation of KIF4A could suppress the colony formation, invasion and migration and cell cycle of OS cells [20, 21]. Here, overexpression of KIF4A polished the effect of DEPDC1 interference to promote the proliferation, invasion and migration of U2OS cells and HUVEC tube formation.

Hippo signaling pathway is a highly conserved evolutionarily that regulates organ size and maintains tissue homeostasis by controlling cell proliferation and apoptosis [29]. In recent years, more and more studies have found that Hippo pathway plays an important role in blander cancer, lung cancer, breast cancer, liver cancer and colorectal cancer [30, 31]. Hsa_circ_0005273 could upregulate the expression of YAP1 through miR-200a-3p, thus promoting the progression of breast cancer [32]. Oncoprotein CagA could promote YAP expression, which promoted the EMT of gastric cancer [33]. Verteporfin (VP), a YAP specific inhibitor inhibited YAP-induced bladder cancer cell growth and invasion [34]. It has been found that Hippo signaling pathway can regulate the proliferation, apoptosis, invasion and metastasis of OS cells [35]. The YAP and TAZ are two important transcriptional co-activators that are negatively regulated by the Hippo signaling pathway. High expression of YAP/TAZ could promote cancer development and inhibition of YAP and TAZ might be useful to treat tumors with high YAP and/or TAZ activity [36]. The present study indicated that down-regulation of DEPDC1 activated the Hippo signaling pathway to overexpress p-LATS1 and p-YAP, thereby inhibiting YAP to suppress the proliferation of OS cells. Also, overexpression of KIF4A could reverse the effect of down-regulation of DEPDC1 on Hippo signaling pathway.

In conclusion, the expression of DEPDC1 and KIF4A was increased in OS cells. Down-regulation of DEPDC1 inhibited the proliferation, invasion and migration of OS cells and HUVECs tube formation through the activation of Hippo signaling pathway, which could be reversed by upregulation of KIF4A. The expression of DEPDC1 and KIF4A in osteosarcoma tissues and the correlation of them will be investigated in future study.

## Data Availability

The datasets used and/or analyzed during the current study are available from the corresponding author on reasonable request.
